# An optimized high-performance liquid chromatography (HPLC) method for benzoylmesaconine determination in *Radix Aconiti Lateralis Preparata *(*Fuzi*, aconite roots) and its products

**DOI:** 10.1186/1749-8546-3-6

**Published:** 2008-05-30

**Authors:** Ying Xie, Hua Zhou, Yuen Fan Wong, Zhongqiu Liu, Hongxi Xu, Zhihong Jiang, Liang Liu

**Affiliations:** 1 School of Chinese Medicine, Hong Kong Baptist University, Hong Kong SAR, PR China; 2 Hong Kong Jockey Club Institute of Chinese Medicine, Hong Kong SAR, PR China

## Abstract

**Background:**

Benzoylmesaconine (BMA) is the main *Aconitum *alkaloid in *Radix Aconiti Lateralis Preparata *(*Fuzi*, aconite roots) with potent pharmacological activities, such as analgesia and anti-inflammation. The present study developed a simple and reliable method using BMA as a marker compound for the quality control of processed aconite roots and their products.

**Methods:**

After extraction, a high-performance liquid chromatography (HPLC) determination of BMA was conducted on a RP-C_18 _column by gradient elution with acetonitrile and aqueous phase, containing 0.1% phosphoric acid adjusted with triethylamine to pH 3.0.

**Results:**

A distinct peak profile was obtained and separation of BMA was achieved. Method validation showed that the relative standard deviations (RSDs) of the precision of BMA in all intra-day and inter-day assays were less than 1.36%, and that the average recovery rate was 96.95%. Quantitative analysis of BMA showed that the content of BMA varied significantly in processed aconite roots and their products.

**Conclusion:**

This HPLC method using BMA as a marker compound is applicable to the quality control of processed aconite roots and their products.

## Background

**Figure 1:**
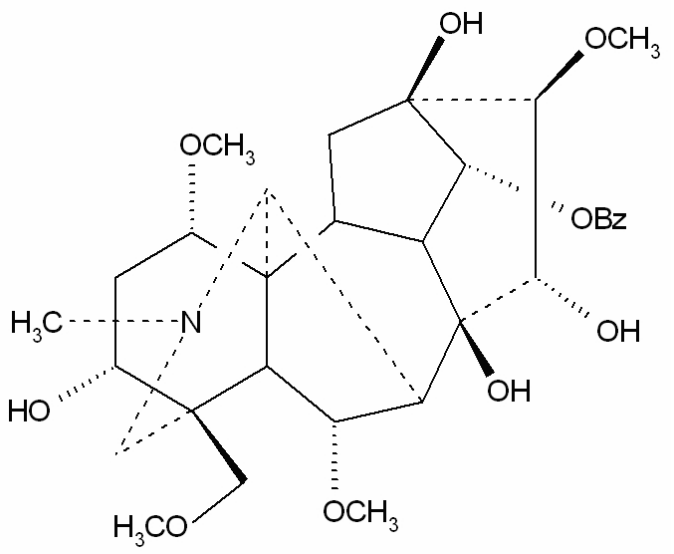
Chemical structure of benzoylmesaconine (BMA).

Plants of the genus *Aconitum *are widely distributed across Asia and North America. For over two thousand years, *Radix Aconiti Lateralis Preparata *(*Fuzi*, aconite roots) has been used in China to relieve joint pain and treat rheumatic diseases [1]. Studies demonstrated some pharmacological effects of aconite roots, such as analgesia and anti-inflammation [2,3]. Processing aconite roots is necessary to remove the poisonous diester-diterpene type *Aconitum *alkaloids including aconitine, mesaconitine and hypaconitine. For example, before used in proprietary herbal products, the toxicity of these alkaloids can be lowered by hydrolysis into much less poisonous benzoylaconines which are the products of deacetylation of the 8β-acetoxyl [4]. Previously, we established a high-performance liquid chromatography (HPLC) method for determining these three toxic *Aconitum *alkaloids [5]. By comparing their peak areas, we found that benzoylmesaconine (BMA, Figure 1), one of the benzoylaconines, was more abundant than the other two alkaloids in most processed aconite roots [5]. It was reported that BMA at the dosage of 30 mg/kg (*per oral*) significantly increased the pain threshold in rats. Its analgesic potency was as potent as that of aconite roots at 1000 mg/kg (*per oral*) [6]. Recently, BMA was further shown to have specific cellular immunological activities, antiviral and antifungal activities [7], antinociceptive effects [8], and stimulating activities on cytokines secretion [9]. Due to its abundance and pharmacology, BMA was selected as a single marker compound for the evaluation of the quality of the processed aconite roots and their proprietary herbal products.

The present study developed a suitable analytical method for identification and quantification of BMA to simplify the quality control procedures. Several HPLC methods have been reported [10], none of which is efficient. For instance, the target compounds could not be efficiently separated in acetonitrile. Tetrahydrofuran (THF) was used as the organic component of the mobile phase, but THF would damage the RP-C_18 _column when its percentage was over 10% [10]. In our previous HPLC method, buffer with a pH value around 10 was used as mobile phase and a special RP-C_18 _column was required to stand such an alkaline buffer (pH > 8) [5,11]. At the moment, gas chromatography-mass spectrometry (GC-MS) and liquid chromatography-mass spectrometry (LC-MS) methods are applicable, but both require sophisticated sample preparation, such as pre-column derivation and solid phase extraction (SPE) [12-14]. In the present study, we developed a simple and reliable HPLC method for quantitative determination of BMA in processed aconite roots and their products.

## Methods

### Processed aconite roots and their proprietary products

Nine batches of the processed aconite roots were purchased from herbal medicine markets in Sichuan, Shanxi and Guangdong provinces in China (Table 1).

**Table 1:** BMA content (μg/g) in processed aconite roots (n = 2)

Samples (batch no)	Sample source	Mean (SD) (μg/g)
FZ040525-s1	Sichuan province	13.4 (0.5)
FZ040525-s2	Sichuan province	33.5 (0.1)
FZ030113-01	Shanxi province	25.3 (0.1)
FZ030616-01	Sichuan province	46.2 (1.2)
FZ030902-01	Guangdong province	10.7 (0.3)
FZ040406-01	Guangdong province	79.1 (1.0)
FZ040406-03	Guangdong province	164.0 (3.6)
FZ040406-07	Guangdong province	2.9 (0.1)
FZ040406-11	Guangdong province	4.6 (0.1)

The Chinese proprietary products containing processed aconite roots used in this study included *Guifulizhong Wan *(GW, Foshan Fengliaoxiang Pharmaceutical Co, Guangdong, China), *Sanqisangyao *Capsule (SC, Guangxi Yulin Pharmaceutical Co, Guangxi, China), *Haimabushen Wan *(HW, Tianjin Zhongxin Pharmaceutical Co, Tianjin, China), and *Jinguishenqi Wan *(JW, Tongrentang Pharmaceutical Co, Beijing, China). The main indications of these products are muscular disorders, joint pain and arthritis. *Qingfuguanjieshu *Capsule (QC), a new antiarthritic herbal preparation being developed in our research group [15,16], was also used in the present study.

### Chemicals and reagents

Acetonitrile was of HPLC grade (International Laboratory, USA). Twenty-eight per cent (28%) ammonia solution (AJAX Chemicals, Australia), 95% ethanol (UNI-CHEM, Hong Kong, China), chloroform (TEDIA, USA), ethyl acetate, triethylamine, 85% phosphoric acid, diethyl ether anhydrous (International Laboratory, USA) and hydrochloric acid (Merck, Germany) were of guaranteed reagent grade. Deionized water was prepared using a Millipore water purification system (Millipore Corp, USA).

The reference standard for BMA was prepared from mesaconitine (National Institute for the Control of Pharmaceutical and Biological Products, China) which was heated at 100°C in dioxane-water (1:1) for 6 hours and purified by column chromatography and crystallization [5,11]. The identity and purity (≥ 95%) of BMA were confirmed by TLC (thin-layer chromatography), HPLC, and spectroscopy (^1^H-NMR and MS) [17].

### HPLC conditions

An Agilent 1100 series LC system (Hewlett Packard, USA) was used in the study, which consisted of a G1311A quaternary pump, a G1322A degasser, a G1315A diode-array detector and a G1313A autosampler.

Alltima RP-C_18 _(ID 250 × 4.6 mm, particle size 5 μm, Alltech Associates, Inc., USA) was used as the stationary phase companied by Alltima RP-C_18 _guard column (ID 7.5 × 4.6 mm). Elution of the alkaloids was carried out using a gradient of acetonitrile (A) and buffer solution (B, containing 0.1% phosphoric acid and 0.1% triethylamine, adjusted to pH 3.0 with triethylamine) at a flow-rate of 1.0 ml/min. The gradient elution of the mobile phase was 13–18 % A in 0–20 min, 18–21 % A in 20–40 min, 21–22 % A in 40–45 min and 22–70 % A in 45–50 min. Detection was carried out at 240 nm with a reference wavelength of 550 nm at room temperature. The injection volume was 20 μl for all HPLC runs.

### Preparation of sample solutions

Processed aconite roots were pulverized into powder, passed through a 0.45 mm sieve, and stored in a desiccator. The powder (1.5 g) was extracted with 50% ethanol three times (4.0 ml, 4.0 ml and 2.0 ml respectively and 60 min each time) by sonication (Branson 5210 Ultrasonicator, Balkowitsch Enterprises, Inc., USA) at room temperature, and then vortexed for 2 min respectively. The mixture was centrifuged for 10 min at 3000 rpm (Eppendorf 5810 Centrifuge, Scios, Inc., USA). The supernatants were combined and transferred to a 10.0 ml volumetric flask, with 50% ethanol making up the volume.

Powdered pills or contents of capsules of the proprietary products (approximately 1.0 g) were accurately weighed. Each sample was dissolved in 10 ml of HCl solution (0.05 M) by sonication for 60 min, and extracted with ethyl acetate three times (10 ml each time). The acidic aqueous solution was separated and basified with 600 μl of 28% ammonia solution and further extracted with 10 ml of mixed solvents of diethyl ether and ethyl acetate (1:1) three times by vortexing for 2 min. The resulting mixtures were centrifuged at 3000 rpm (Eppendorf 5810 Centrifuge, Scios Inc, USA) for 5 min and the combined supernatants were evaporated to dryness under air stream. The residue was dissolved in 1.0 ml of HCl solution (0.01 M) by sonication for 1 min.

### Preparation of standard solutions

BMA was accurately weighed and dissolved in HCl solution (0.01 M) to produce a stock standard solution at a final concentration of 0.4084 mg/ml. This stock solution was used to prepare standard solutions for method validation and calibration curves. The standard solutions were stored at 4°C and remained stable for at least one month. Calibration curves were established at seven concentration (μg/ml) points of 4.08, 10.21, 20.42, 40.84, 81.68, 122.52 and 204.20 respectively. For recovery test, a standard solution was prepared at a concentration of 12.25 μg/ml.

### Validation of the HPLC method

#### Precision

Expressed as relative standard deviations (RSDs), precision was evaluated by HPLC runs with standard solutions at three concentrations under the optimal condition five times in one day for intra-day variation test and twice a day on three consecutive days for inter-day variation test.

#### Repeatability

Six aliquots of QC were conducted for repeatability test. The processing of the aliquots followed the same method described in the section of preparation of sample solutions. The RSDs of the six aliquots were calculated for evaluation of repeatability.

#### Recovery

HCl solution (10 ml, 0.05 M) was added to accurately weighted QC (approximately 0.1 g) in which the content of BMA was known. The sample solution was spiked with 1000 μl of the standard solution of BMA for recovery test. The processing of the prepared samples (n = 6) followed the method described in the section of preparation of sample solutions.

### Statistical analysis

Data analysis, including calculation of standard deviation (SD) and linear regression, was conducted using Excel 2003 (Microsoft, USA). P values less than 0.05 were considered as statistically significant.

## Results and discussion

### Optimization of HPLC conditions

**Figure 2:**
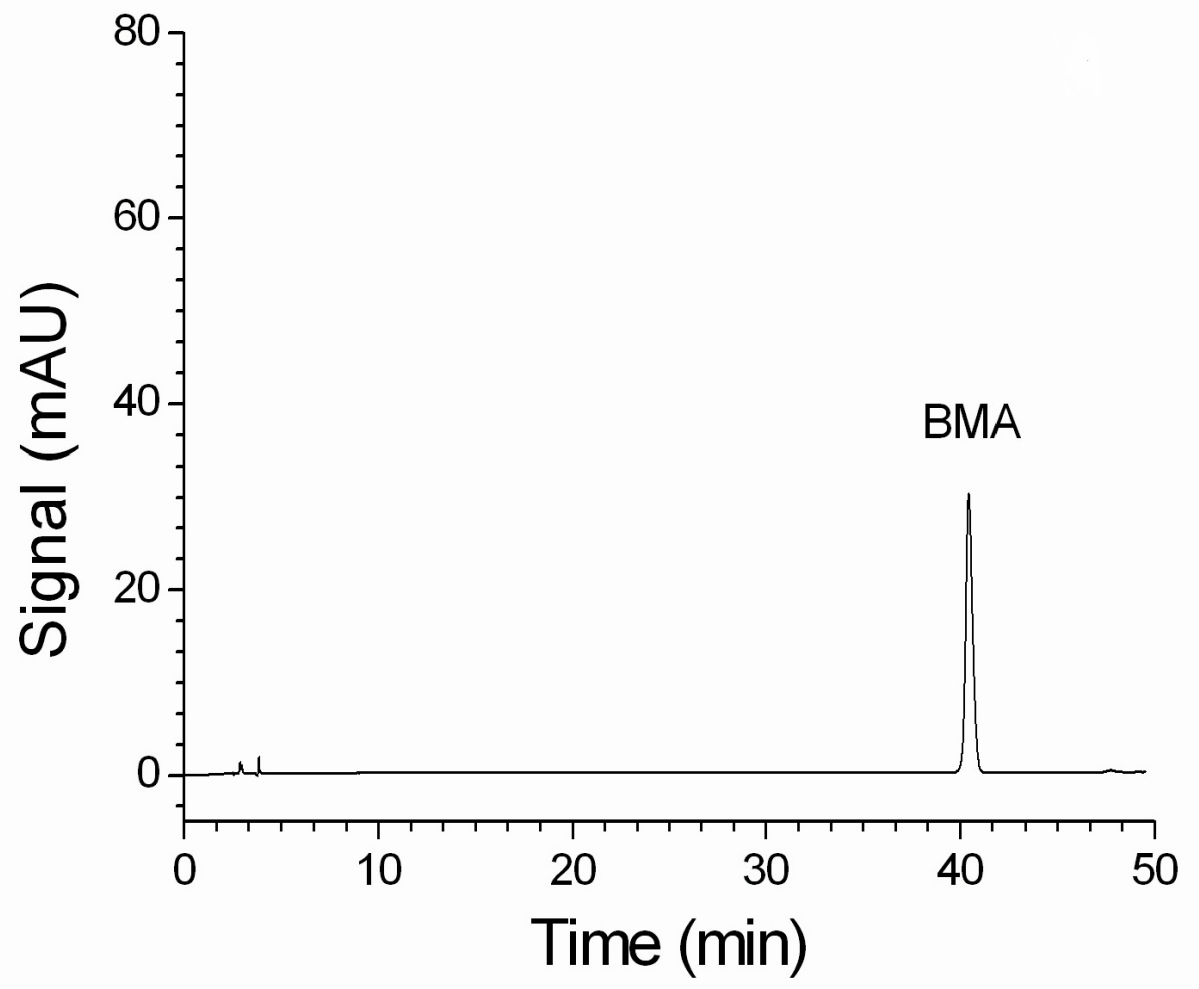
An HPLC chromatogram of BMA standard solution.

**Figure 3:**
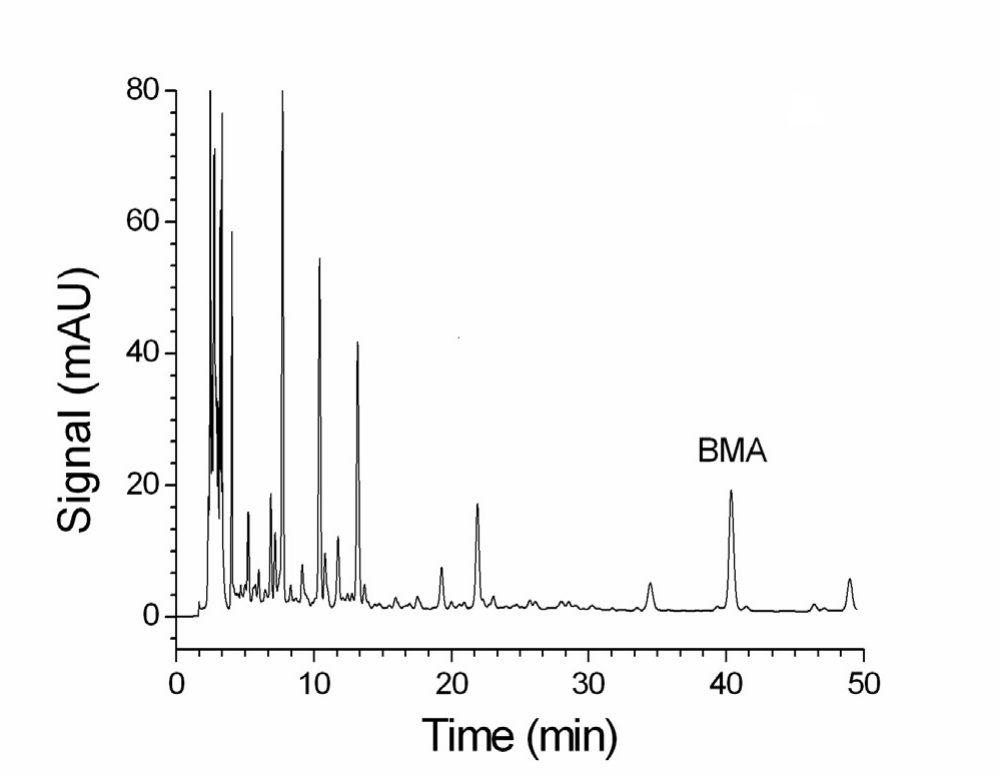
An HPLC chromatogram of BMA in processed aconite roots (batch no 040406-01).

**Figure 4:**
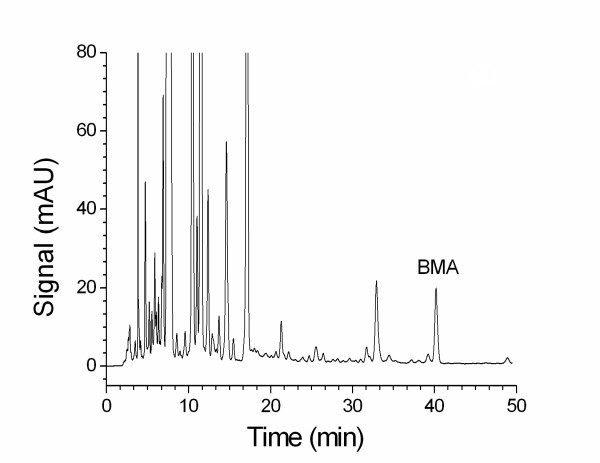
An HPLC chromatogram of BMA in QC.

The use of phosphoric acid (0.1 %) with triethylamine as the mobile phase produced better symmetry of the peak in the chromatogram. A gradient elution program was developed to isolate BMA and to determine the content of BMA in the proprietary products. Figure 2 shows a chromatogram of BMA (35 μg/ml) in standard solution. Figure 3 shows a chromatogram (detection at 240 nm) of BMA in 50 % ethanol extract of processed aconite roots. The results indicate that the retention time (43.5 min) of BMA in processed aconite roots (Figure 3) and in QC preparation (Figure 4) was consistent with that of BMA in standard solution (Figure 2).

**Figure 5:**
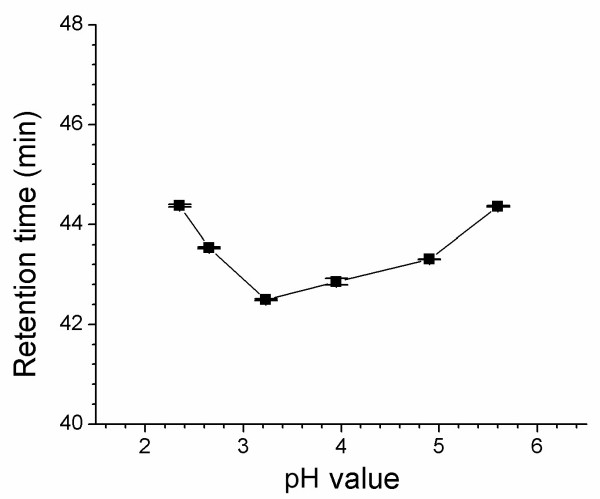
Relation of pH and the retention time of BMA.

By adjusting the triethylamine content to obtain the pH values from 2.3 to 5.6, we investigated the pH dependence of the retention time. The results show BMA retention time remained relatively stable in the range of 42–44 min as pH value increased (Figure 5). Due to a long elution time and drifting baseline at pH values below 2.6 and above 4.9 respectively, the HPLC runs in the present study were carried out at pH 3.0 for best effect.

### Optimization of extraction conditions

Common extracting solvents including water and aqueous ethanol (95 %, 75 %, 50 % and 25 %) were evaluated for their efficiency in extracting BMA from processed aconite roots. Since BMA is an alkaloid, an acidic extraction solvent (0.01 M HCl) was also tested. The results show that the extraction efficiency of these solvents was in the following order: 50 % ethanol > 25% ethanol > 0.01 M HCl > 75 % ethanol > 95 % ethanol. For highest efficiency, 50 % ethanol was selected for extracting BMA in processed aconite roots in the present study (Table 2).

**Table 2:** Efficiency of different solvents in extracting the content of BMA (μg/g) from processed aconite roots (n = 2)

Solvents	Mean (SD) (μg/g)	RSD (%)
0.01 M HCl	20.8 (0.3)	1.24%
25% ethanol	22.7 (0.0)	0.10%
50% ethanol	25.3 (0.1)	0.45%
75% ethanol	14.8 (0.3)	0.18%
95% ethanol	0.55 (0.06)	10.98%

Note: Sample FZ030113-01 was used in this experiment.

As a single-step extraction with 50 % ethanol contained many impurities, we developed a multi-step extraction to remove some non-alkaloid components in the extracts. Powdered proprietary products were dissolved in 10 ml of HCl solution (0.05 M) by sonication for 60 min, which were subsequently extracted with ethyl acetate for three times (10 ml each time). The acidic aqueous solutions were then basified with 600 μl of 28 % ammonia solution and further extracted with 10 ml of mixed solvents of diethyl ether and ethyl acetate (1:1) for three times. The recovery rate of BMA was 90.64% (SD 0.58%) when diethyl ether was the extraction solvent. The recovery rate was nearly perfect when ethyl acetate was the extraction solvent; however, there was a small interference peak at the retention time of 43 min. Thus, a mixed solvent of diethyl ether and ethyl acetate (1:1) was used, whereby the recovery rate was 96.95% (SD 1.01%) and no interference peak was observed.

### Validation of the HPLC method

**Figure 6:**
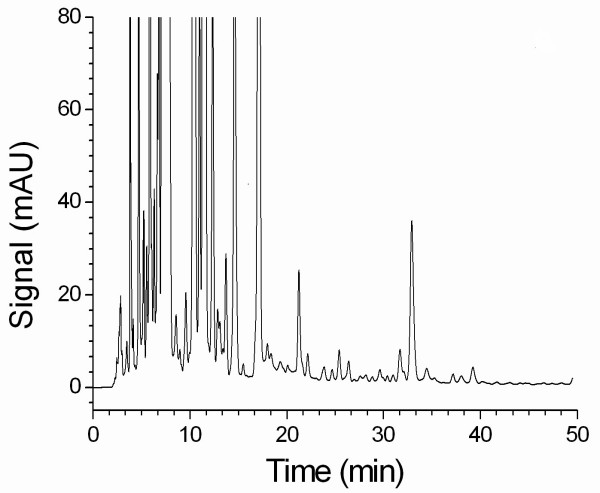
An HPLC chromatogram of QC product without processed aconite roots (negative control).

The BMA peaks in processed aconite roots and in their proprietary products were identified through comparison of the retention times and UV spectra with those of the reference standards. Peak purity was confirmed by data from a photodiode array detector (DAD). Furthermore, a comparative study was carried out with QC samples and QC without processed aconite roots (negative control). There is no peak corresponding to BMA in the chromatogram of the negative control (Figure 6).

The linearity of the BMA concentrations (μg/ml) versus peak areas was investigated in the range of 4.08–204.20 μg/ml; the result is expressed as the value of the coefficient of determination (R^2^). The following equation was obtained: Y = 18.518X + 5.651 (R^2 ^= 0.9998, P < 0.001).

The limit of detection, defined as the amount of the compounds needed to produce a signal (S) at least three times larger than noise (N), was determined to be 8 ng for BMA with an injection volume of 20 μl.

The precision of the intra-day (five times per day) and inter-day (twice a day for three consecutive days) data was indicated by RSDs which were less than 1.36% for BMA at three concentrations (Table 3). These results suggest that BMA is stable in acidic solution (0.01 M HCl). The accuracy was determined by back calculation of precision samples at three concentrations against a calibration curve prepared each day.

**Table 3:** Precision of the intra-day and inter-day measurements

Conc. μg/ml	Intra-day ^a^			Inter-day ^b^		
	Peak areas Mean (SD)	RSD %	Precision %	Peak areas Mean (SD)	RSD %	Precision %
Low (4.08)	63.0 (0.8)	1.36	95.90%	65.3 (0.5)	0.88	95.08%
Mid (20.42)	303.1 (0.4)	0.15	95.48%	309.9 (1.8)	0.60	95.80%
High (122.52)	1870.0 (7.5)	0.39	97.55%	1890.0 (17.8)	0.95	98.59%

Note: ^a^: n = 5; ^b^: n = 6 (twice a day for three consecutive days).

The RSDs of the repeatability test were less than 0.29% for BMA at 132.8 μg/g (SD 0.4 μg/g) in the QC samples (n = 6). The average recovery rate obtained was 96.95% (SD 1.01%) with RSDs of 0.01% (n = 6). This chromatographic system is suitable for quantitative determination of BMA in processed aconite roots and their proprietary products.

### Quantitative determination of BMA

The content of BMA in the five proprietary products, and nine batches of processed aconite roots were determined using the established HPLC method described above. The content of BMA (Tables 1 and 4) was calculated with the regression equations obtained from the calibration curves.

**Table 4:** BMA content (μg/g) in five herbal proprietary products containing processed aconite roots (n = 2)

Product	Batch no	Dosage (g/day)	Mean (SD) (μg/g)
GW	0405005	18	6.9 (0.1)
SC	20030801	2.25	245.0 (0.7)
HW	030769	5.4	58.0 (0.2)
JW	3030904	10	6.0 (0.0)
QC	20040901	8.5	132.8 (0.4)

Note: GW: *Guifulizhong Wan*, SC: *Sanqisangyao *Capsule, HW: *Haimabushen Wan*, JW: *Jinguishenqi Wan*, QC: *Qingfuguanjieshu *Capsule

Significant variations in terms of BMA content were found among different batches of processed aconite roots and among different proprietary products (Tables 1 and 4). Differences in species, places of origin, processing methods of the raw materials and pharmaceutical manufacturing processes may all contribute to the variations.

As herbal products containing processed aconite roots are increasingly used for anti-inflammation and analgesia, simple and reliable methods for quality control of these products are urgently needed. It was reported that BMA was effective in anti-inflammation and analgesia in animals [2,6]. Thus, BMA was selected as a marker compound to assess the quality of processed aconite roots and their proprietary products.

In the present study, extraction method was optimized. Results from the recovery test show that the use of two solvents is better than that of a single solvent in preparing samples of proprietary products.

In the analysis of *Aconitum *alkaloids using a reversed phase C_18 _HPLC column, tailing peaks are usually caused by the retention effects of free silanol groupings. Instead of using THF as an organic solvent or basic buffer in the mobile phase, we used triethylamine to improve separation and peak shapes. Moreover, this HPLC method should be easily repeated in other labs. The present study also indicates that the retention volumes of BMA remained relatively unchanged over an acidic pH region (Figure 5).

## Conclusion

Method validation data indicate that the developed HPLC method as described in this paper is reliable, reproducible and accurate for the determination of BMA in processed aconite roots and their products. The results also show that the content of BMA varied significantly in different batches of the processed aconite roots including five proprietary products. This method is suitable for routine assessment of the quality of processed aconite roots and their products.

## Abbreviations

BMA: benzoylmesaconine; DAD: photodiode array detector; GC-MS: gas chromatography-mass spectrometry; HPLC: high-performance liquid chromatography; LC-MS: liquid chromatography-mass spectrometry; RSDs: relative standard deviations; SD: standard deviation; SPE: solid phase extraction; THF: tetrahydrofuran

## Competing interests

The authors declare that they have no competing interests.

## Authors' contributions

YX contributed to the conception and design of the study, carried out the experimental work, and drafted the manuscript, ZJ contributed to the design of the study and helped revise the manuscript, HZ participated in the design of the study and performed statistical analysis, YW and ZL participated in the experimental work of *Qingfuguanjieshu *Capsule and helped with data acquisition, HX contributed to the design of the study, LL, the principal investigator of the project, contributed to the conception and design of the study, revised and finalized the manuscript. All authors read and approved the final version of the manuscript.
